# Exertional Heat Illness Preparedness Strategies: Environmental Monitoring Policies in United States High Schools

**DOI:** 10.3390/medicina56100486

**Published:** 2020-09-23

**Authors:** Samantha E. Scarneo-Miller, Luke N. Belval, Susan W. Yeargin, Yuri Hosokawa, Zachary Y. Kerr, Douglas J. Casa

**Affiliations:** 1 Division of Athletic Training, School of Medicine, West Virginia University, Morgantown, WV 26508, USA; 2 Institute for Exercise and Environmental Medicine, University of Texas Southwestern and Texas Health Resources Presbyterian Hospital Dallas, Dallas, TX 75231, USA; LukeBelval@texashealth.org; 3 Department of Exercise Science, Arnold School of Public Health, University of South Carolina, Columbia, SC 29208, USA; SYEARGIN@mailbox.sc.edu; 4 Faculty of Sport Sciences, Waseda University, Saitama 359-1192, Japan; yurihosokawa@waseda.jp; 5 Department of Exercise and Sport Science, University of North Carolina-Chapel Hill, Chapel Hill, NC 27599, USA; zkerr@email.unc.edu; 6 Korey Stringer Institute, Department of Kinesiology, University of Connecticut, Storrs, CT 06269, USA; douglas.casa@uconn.edu

**Keywords:** environment, exertional heat illness, policies and procedures, health behavior

## Abstract

*Background and objectives:* Environmental monitoring allows for an analysis of the ambient conditions affecting a physically active person’s ability to thermoregulate and can be used to assess exertional heat illness risk. Using public health models such as the precaution adoption process model (PAPM) can help identify individual’s readiness to act to adopt environmental monitoring policies for the safety of high school athletes. The purpose of this study was to investigate the adoption of policies and procedures used for monitoring and modifying activity in the heat in United States (US) high schools. *Materials and Methods:* Using a cross-sectional design, we distributed an online questionnaire to athletic trainers (ATs) working in high schools in the US. The questionnaire was developed based on best practice standards related to environmental monitoring and modification of activity in the heat as outlined in the 2015 National Athletic Trainers’ Association Position Statement: Exertional Heat Illness. The PAPM was used to frame questions as it allows for the identification of ATs’ readiness to act. PAPM includes eight stages: *unaware of the need for the policy, unaware if the school has this policy, unengaged, undecided, decided not to act, decided to act, acting,* and *maintaining.* Invitations were sent via email and social media and resulted in 529 complete responses. Data were aggregated and presented as proportions. *Results:* Overall, 161 (161/529, 30.4%) ATs report they do not have a written policy and procedure for the prevention and management of exertional heat stroke. The policy component with the highest adoption was modifying the use of protective equipment (acting = 8.2%, maintaining = 77.5%). In addition, 28% of ATs report adoption of all seven components for a comprehensive environmental monitoring policy. *Conclusions:* These findings indicate a lack of adoption of environmental monitoring policies in US high schools. Secondarily, the PAPM, facilitators and barriers data highlight areas to focus future efforts to enhance adoption.

## 1. Introduction

The increasing frequency, duration and intensity of heatwaves (periods where environmental temperature is excessively high) in the United States (US) will directly impact athletes’ ability to participate in sports safely [1]. Concomitant with more severe environmental conditions, the incidence of exertional heat illness has also increased, illustrating the compulsory need for healthcare practitioners to intervene and prevent these potentially fatal conditions [2,3]. In the US, it is estimated that 9000 exertional heat illness’ occur in high school sports yearly [4]. Environmental monitoring is essential to prevention efforts, as it can help to determine when changes to exercise sessions are needed, which can consequently minimize risk of exertional heat illness [5,6].

Because human tolerance to hot environments is dependent on several factors (e.g., ambient temperature, humidity, solar radiation), best practices for environmental monitoring have supported metrics that are a combination of measurements [5,6]. The gold standard measurement in the sports medicine community is the wet bulb globe temperature (WBGT), which combines a wet bulb, dry bulb and black globe temperature to account for the thermal strain a given environment imposes upon an exercising individual [7]. Previous research has found WBGT to be superior to other indices such as heat index [8], as the WBGT is comprehensive and inclusive of the aforementioned components. Additionally, modifying the amount of protective equipment worn during physical activity can reduce the thermal strain experienced by athletes [9]. Therefore, in conjunction with environmental control, modification of the protective equipment should be considered accordingly. The ability to adequately measure the environmental strain on exercising individuals allows healthcare professionals, coaches, athletes and parents to modify activity in the heat to mitigate exertional heat illnesses.

The National Athletic Trainers’ Association Position Statement: Exertional Heat Illnesses recommends the development of “event guidelines formulated for hot, humid weather conditions based on the type of activity and wet bulb globe temperature (WBGT) [10].” These recommendations include suggested ranges of WBGTs and graduated modifications to activities based upon those measurements. Such measurements should also be taken on-site relative to the specific event, as local measurements of WBGT can vary significantly [11,12]. Additionally, climatological-region-referenced recommended ranges (i.e., northern US, middle US, southern US, as delineated by Grundstein et al. [13]) for these WBGT values have been proposed to reflect differences in typical environmental conditions experienced across the US [13]. These policy recommendations are reiterated through statements by the American College of Sports Medicine [14] and reflect similar guidelines in the US military and occupational safety communities [5].

While the above-described best practice recommendations reflect a significant body of knowledge both in laboratory and field settings, relatively little is known about whether secondary-school athletic trainers (ATs) are implementing environmental monitoring policies effectively. Looking more broadly, ATs appear receptive to the adoption of best practices, as investigations into emergency action plans [15,16] and heat acclimatization policies [17] have yielded mostly encouraging findings with regards to overall policy adoption. However, upon close evaluation, certain aspects of implementing these best practices are limited in their scope. For example, Kerr et al. found that while a large proportion of ATs reported compliance with most aspects of NATA guidelines for heat acclimatization, only 3.9% reported full compliance [17]. The reasons for these deficits and the steps necessary to rectify them are highlighted by evidence supporting the effectiveness of these policies in minimizing the risk of exertional heat illnesses [3].

**Figure 1:**
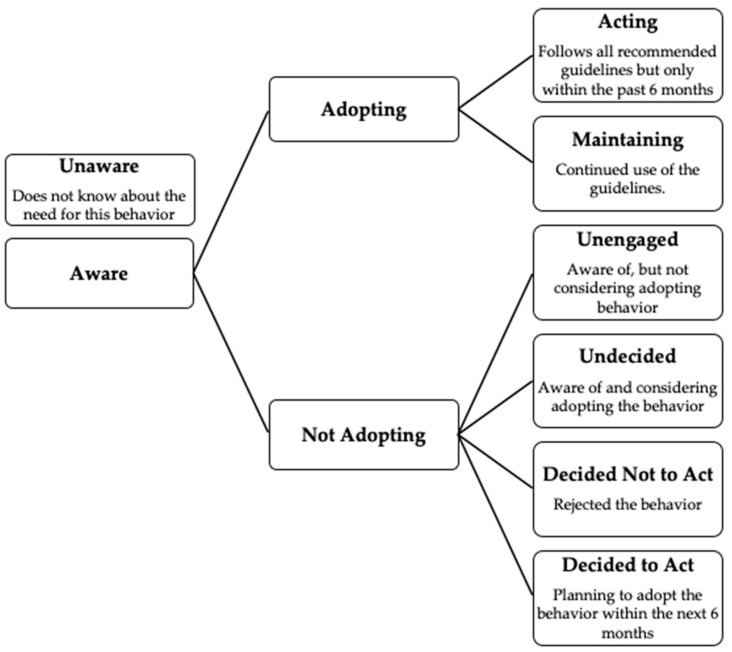
Precaution adoption process model (PAPM) stages.

Furthermore, we contend that it is vital to understand the facilitators that allow ATs to incorporate these policies and the barriers that prevent them. In previous investigations, the precaution adoption process model (PAPM) has been employed to evaluate the readiness of ATs to adopt best practices (Figure 1) [18]. In evaluations of similar athletic emergency policies, ATs have reported support from administration, having themselves present and state requirements as facilitators to implementing best practices, while budgetary constraints were persistently reported as a barrier [19]. Notably, in a study of state high school athletic associations, 53% of these associations required their member schools to develop environmental monitoring policies [20].

To better assist ATs and administrators in adopting and implementing best practice environmental monitoring and modification policies, additional research is needed to determine current practices and their willingness to change policies. Therefore, the purpose of this study was to investigate the adoption of policies and procedures used for monitoring and modifying activity in the heat. Additionally, we sought to evaluate the facilitators and barriers that influenced ATs in implementing these policies. We hypothesized that ATs adopting overall environmental monitoring policies would be higher in prevalence in states that required them.

## 2. Materials and Methods

This study was classified as exempt by the University of Connecticut Institutional Review Board. Using a cross-sectional design, we distributed an online questionnaire to ATs working in high schools in the US. Though secondary school is a more common term internationally, we will use the term ‘high school’ for two reasons: (1) we asked ATs about their high schools and aim to be consistent with the questionnaire terminology; and (2) high school in the US is defined as Grades 9–12 which is the population studied (rather than secondary school, which is often defined as Grades 6–12). It is worth noting that in the US, most serious athletics programs are at the high-school level, rather than general secondary-school level. ATs were asked to report their adoption of environmental related policies.

### 2.1. Participants and Recruitment

ATs were recruited to participate in this study through two primary methods: First, ATs who completed the athletic training locations and services (ATLAS) project were sent email invitations for participation [21]. Second, an online flier was distributed through social media (Facebook, Twitter, Instagram). The use of social media to invite participants to complete the questionnaire has been previously used [22]. An online questionnaire developed through Qualtrics LLC. (Provo, UT, USA) was distributed and posted on social media sites in fall 2018 and spring 2019.

A completed questionnaire consisted of: (1) providing consent to participate; (2) being an AT working in the high school setting; and (3) completing at least 80% of the questions in the questionnaire. A total of 417 ATs completed the questionnaire in fall 2018 and 324 completed the questionnaire in spring 2019, resulting in a total of 741 responses. In the fall 2018, two participants did not answer the first question, “Does your school have a written policy for exertional heat illness (prevention and treatment)?” Therefore, they were removed from the analysis, resulting in 415 fall responses. As this study is part of a larger study, those individuals who had longitudinal data (i.e., completed the questionnaire in both fall and spring) were excluded in the analyses, leaving only those who took it in the fall only or spring only. Given the mixed recruitment methods and the inability to identify how many people saw the social media recruitment posts, we are unable to calculate a proper response rate.

### 2.2. Questionnaire

An online questionnaire (Supplementary Materials) was developed based on position statements, consensus statements and inter-association task force documents outlining best practice standards for monitoring the environment [10,23,24]. ATs were first asked to report their characteristics (age, students enrolled in their high school, years in the profession, years at their high school overall). Subsequent questions included ATs reporting the category that best describes their high school’s written policy based on the PAPM stages. The PAPM is a health behavior model that aims to identify an individual’s readiness to act [25]. In this case, we aimed to identify readiness to act as it relates to environmental monitoring policies and procedures. Traditionally, there are seven stages of the PAPM: *unaware*, *unengaged*, *undecided*, *decided not to act*, *decided to act*, *acting*, *maintaining*. In this investigation, we split *unaware* into two separate stages: *unaware of the need for the policy* and *unaware if the school has this policy.* Then, ATs were asked to select the category that best describes their high school’s written policies and procedures on exertional heat illness (prevention and treatment). If ATs reported *acting* or *maintaining* they were shown 6 additional questions on the modification of activity in the heat. If ATs reported *unaware*, *unengaged*, *undecided*, *decided not to act or decided to act* they were not shown those additional questions as they did not have a written policy. Two additional questions about best practices were asked regarding the primary person overseeing the process for heat modification and how modifications for activity in the heat is determined. Finally, participants were asked to select all that apply for facilitators and barriers to the development of a comprehensive heat modification policy from a comprehensive list of predetermined choices.

The questionnaire was developed by content area experts (S.E.S.-M., L.N.B., S.W.Y., Y.H., Z.Y.K., D.J.C.) and distributed to seven high school ATs for pilot testing. The pilot test identified if the questions were clear, important and relevant to the ATs. Changes to the questionnaire were made following feedback from the pilot testing.

### 2.3. Data Analysis

Data were aggregated from fall 2018 and spring 2019. Descriptive statistics (i.e., frequencies and percentages) provided the proportions of responses for each question. PAPM questions were dichotomized into “Adopting” (e.g., yes policy) which included *acting* or *maintaining* or “not adopting” (e.g., no policy) which included *unaware*, *unengaged*, *undecided*, *decided not to act or decided to act*. Next, for the dichotomized variable from the question “my high school’s written policy is based on environmental conditions measured by an on-site WBGT,” we ran independent t-tests to examine whether differences existed between mean age and the mean number of students enrolled. For the categorical data on the ATs’ years in their professional role overall along with years in their high school, we created dichotomous variables and conducted chi-squared tests (χ^2^) with the same question (measured by an on-site WBGT). Years in professional role was dichotomized into 0–10 years (56.4%) vs. 11+ years (43.6%) whereas years in their high school was dichotomized into 0–5 years (60.2%) vs. 6+ years (49.8%). The cutoff values were determined based on group distribution. Finally, prevalence ratios (PRs) with 95% confidence intervals (95% CI) were calculated to identify differences between PAPM stages. PRs were conducted to compare those in the *maintaining* stage who did not report barriers to a comprehensive heat policy v. those in the *maintaining* stage who reported barriers to a comprehensive policy (i.e., barriers vs. no barriers compared with stage). Of note, facilitators and barriers were offered to the participants to “select all that apply” from a predetermined list. At least one participant selected each of the predetermined selections.

## 3. Results

Participant demographics are reported in Table 1.

### 3.1. Adoption

A total of 161 ATs reported they did not have a written policy and procedure for the prevention and management of exertional heat stroke (*n* = 161/529, 30.4%) (Table 2). Overall, the policy component with the highest adoption was modifying the use of protective equipment (*acting* = 8.2%, *n* = 30 *maintaining* = 77.5%, *n* = 282) (Table 3). No significant differences in ATs’ age or the number of students enrolled were found between ATs reporting that their schools had or did not have a written policy to measure on-site WBGT (age *p* = 0.57, students *p* = 0.11). In addition, no significant differences in the distributions of having versus not having a written WBGT policy by years in profession (0–10 years v 11+ years; χ^2^ = 0.01, *p* = 0.913) or years at their current high school (0–5 years vs. 6+ years; χ^2^ = 0.34, *p* = 0.556).

Regarding the PAPM responses, the question resulting in the most variability in answers was, “Is your written policy based on environmental conditions measured by an on-site wet bulb globe thermometer?” (*unaware needed* = 3.6%, *unaware if have* = 6.9%, *decided not to act* = 9.9%, *unengaged* = 8.2%, *undecided* = 9.9%, *decided to act* = 4.4%, *acting* = 8.8%, *maintaining* = 48.4%).

**Figure 2:**
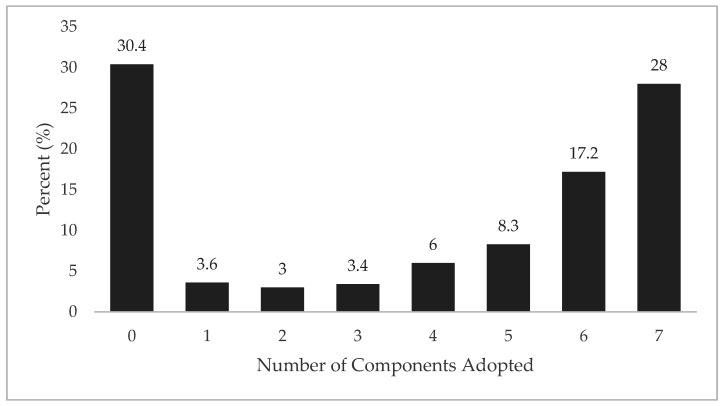
Number of components adopted (%).

**Figure 3:**
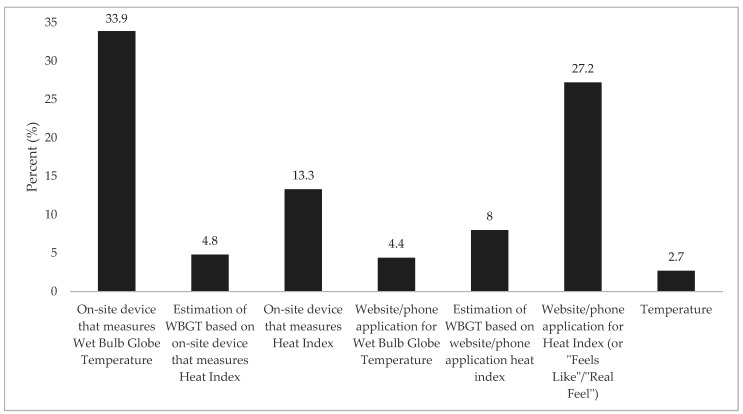
Responses from question “Our school modifies activities based on...” (%).

Overall, 28% of ATs in this sample reported adopting all seven evidence-based components for environmental monitoring and modification (Figure 2). When asked through what methods do their schools modify activities in the heat, over 1/3 of ATs (33.9%) reported using an on-site device that measures WBGT (Figure 3). When asked about the primary person who oversees the process (checks environmental conditions each day, informs coaches, etc.) to ensure that the progression is followed, the AT was the most common person (85%, *n* = 452), followed by: athletic director (35.7%, *n* = 190), head coach (15.6%, *n* = 83), assistant coach (3.6%, *n* = 19), principal/head master (3.6%, *n* = 19), vice principal/assistant head master (2.1%, *n* = 11), nurse (1.95, *n* = 10) and strength and conditioning coach (0.9%, *n* = 5).

### 3.2. Facilitators and Barriers for Adoption of a Comprehensive Environmental Monitoring Policy

The most common facilitators included support from someone in an authoritative position (56%, *n* = 298), state mandate from the high school athletics association (49.1%, *n* = 261) and state legislation to mandate this policy (49.1%, *n* = 261) (Table 3). Overall, 43.6% (*n* = 232) of ATs reported no barriers encountered. However, the barrier most commonly reported was resistance or apprehension from head coaches to modify practices (33.6%, *n* = 179) (Table 4). Compared to ATs who reported barriers, those ATs not reporting barriers were more likely to report *maintaining* a written policy for the prevention and management of exertional heat stroke (73.2% vs. 53.7%; PR = 1.36, 95% CI = 1.20, 1.55).

## 4. Discussion

Environmental monitoring and activity modifications in the heat to reduce the risk of EHS have been part of recommended policy and procedures by the National Athletic Trainers’ Association since 2002 [26]. However, our data revealed 30.4% of ATs in this study had not yet adopted written policies and procedures for the prevention and management of EHS. Further, use of on-site WBGT meters demonstrated the lowest rate of adoption (57%), which is of concern as there are limitations in relying on other environmental metrics (e.g., Heat Index, air temperature, humidity) that do not take into account solar heat gain and discrepancies between on-site measurements and data obtained from the nearest weather station [11,27]. These limitations could mislead the timing of activity modification implementation and may affect the effectiveness of environmental heat stress related policies.

This study also used the PAPM framework (*unaware of the need for the policy*; *unaware if the school has this policy*; *unengaged*; *undecided*; *decided not to act*; *decided to act*; *acting*; *maintaining*) to stratify high school ATs’ readiness to adopt environmental monitoring policies and procedures (Table 2). While at least half of the participants indicated the adoption of best practice standards (e.g., *acting* or *maintaining*) in all seven components (range, 57.2–85.7%), a majority of the non-adoption participants did *not* select *decided to act*, indicating these individuals do not have intentions to adopt these standards in the near future (Table 2). Compared to other standards, the use of on-site WBGT meters had high rates of *decided not to act* and *undecided* responses, suggesting that these ATs are either unfamiliar about the advantages of taking on-site WBGT or have limited support from other stakeholders of athletics program to adopt the policy. Our data support these hypotheses as the top two barriers in adopting a comprehensive heat modification policy were (1) no barriers encountered and (2) resistance or apprehension from head coaches to modify practices. It is plausible those participants who responded, “no barriers encountered,” yet do not adopt these best practices, may lack the knowledge on the topic to be able to identify those barriers they may have encountered if they were to adopt these policies in their high schools.

These barriers may be overcome by shifting belief among ATs and head coaches with systemic intervention, such as a state mandate and tailored education to reassure them that the use of on-site WBGT and other environmental heat stress best practice standards will not result in inequality in practice and competition times. Further, demonstrating the need for heat policies, regardless of the geographical location of the school may facilitate change. Though Cooper et al. have shown that WBGT >28 °C is associated with greater incidence of exertional heat illness among collegiate football players across the contiguous US [28], retrospective analysis of EHS related fatalities in football players in another study [29] demonstrated that the risk was heightened when the observed WBGT was unexpectedly high based on the local climate. As such, Grundstein et al. proposed a regional heat safety threshold that accounts for variations in climate pattern observed across the contiguous US [13]. The proposed regional adjustments resulted in raising the activity modification threshold for regions that are traditionally hot, ensuring that athletes could still participate in physical activities with proper risk mitigation measures, while detecting unusually warm days in traditionally cool regions by lowering their activity modification thresholds [13]. While the purpose of this current study was not to evaluate the regional differences across the nation, understanding regional guidelines and regional differences in the US may help to perpetuate the shift in the belief among ATs and head coaches for the use of on-site and regionally specific guidelines at their schools.

In this study, 28% of participants met all seven best practice standards. This was higher than the full compliance rate reported by Kerr et al. for ATs’ compliance with the preseason heat-acclimatization guidelines (3.9%) [17]. These differences may be explained by the required actions needed to meet these guidelines. For example, environmental monitoring is often an assumed role of an AT as part of their daily routine for risk mitigation and injury prevention; whereas, components of heat-acclimatization guidelines involve changes in the delivery of practice and training sessions, which is primarily under the provision of the coaches. In the small sample that had full compliance in preseason heat-acclimatization guidelines, a state mandate was mentioned as one of the key facilitators for policy adaption, which supports the strength of systemic intervention as a facilitator for policy adoption [3].

There are several items that need to be addressed in understanding the limitations of this study. First, our questionnaire focused on school policy and procedures with the aim to investigate the adoption of environmental heat stress best practice standards. Consequently, there may be schools that do not have a written policy, but still implements the recommended standards, which we could not capture using the current design. It is important to note the difference between adoption and implementation. Adoption refers to the initiation or delineation of the policy, whereas implementation refers to how the standards are delivered. For example, an AT (athletic trainer) may report they have a policy, but when a policy is evaluated on-site, it may be found that they do not have all of the components required for a comprehensive policy. Future research should aim to explore the differences between ATs’ report of adoption vs. implementation on-site. Second, as with any questionnaire, we assume bias inherent to self-report data (e.g., recall bias, social desirability bias, etc.) in our participants responses. It is also acknowledged within questionnaire designed investigations that those who may be adopting the best practice, may be those more likely to respond to the questionnaire, thus introducing response bias. Third, the questions were phrased to inquire about the school’s decision to use these best practices. As such, the health behavior stages the participant reported may be reflective of the participant’s perception of the school’s behavior towards the best practice, rather than their own behaviors. Future research should aim to differentiate the readiness to act of stakeholders within the school community and ATs’ readiness to act.

## 5. Conclusions

The importance of reducing exertional heat illness’ in high school sport cannot be understated. Environmental monitoring allows for the analysis of the conditions that may affect the body’s ability to thermoregulate properly. The results of this study indicate that nearly 1/3 of the responding ATs were not adopting a policy for the prevention of exertional heat illnesses. Furthermore, when a policy is adopted, it is often not comprehensive or inclusive of the best practices outlined in the National Athletic Trainers’ Association Position Statement. The lack of comprehensive and evidence-based policies and procedures leaves athletes vulnerable to injury and possibly death. These data demonstrate the need for the development of tailored interventions to address adoption of exertional heat illness prevention policies.

## Figures and Tables

**Table 1:** Demographics. The grey coloring was to signify a new column with new state names.

**Age**	35 ± 9 years	**Students**	1297 ± 1044 students
**Years in Profession**	**Years at High School**		
Less than 1 year	5 (0.9)	Less than 1 year	30 (5.7)
1–5 years	169 (31.9)	1–5 years	288 (54.5)
6–10 years	125 (23.6)	6–10 years	95 (18)
11–15 years	73 (13.8)	11–15 years	53 (10)
15 or more years	158 (29.8)	15 or more years	61 (11.7)
**States**			
AL	7 (1.3)	MS	2 (0.4)
AR	6 (1.1)	MT	6 (1.1)
AZ	8 (1.5)	NC	31 (5.8)
CA	26 (4.9)	NE	5 (0.9)
CO	7 (1.3)	NH	2 (0.4)
CT	5 (0.9)	NJ	24 (4.5)
DC	1 (0.2)	NM	6 (1.1)
FL	28 (5.3)	NV	2 (0.4)
GA	16 (3)	NY	12 (2.3)
HI	3 (0.6)	OH	23 (4.3)
IA	6 (1.1)	OK	6 (1.1)
ID	3 (0.6)	OR	8 (1.5)
IL	10 (1.9)	PA	23 (4.3)
IN	19 (3.6)	RI	2 (0.4)
KS	2 (0.4)	SC	12 (2.3)
KY	5 (0.9)	TN	9 (1.7)
LA	10 (1.9)	TX	59 (11.1)
MA	13 (2.4)	UT	6 (1.1)
MD	10 (1.9)	VA	24 (4.5)
ME	5 (0.9)	VT	2 (0.4)
MI	15 (2.8)	WA	15 (2.8)
MN	7 (1.3)	WI	12 (2.3)
MO	10 (1.9)	WY	3 (0.6)
		Not reported	16 (3.0)

**Table 2:** Policy adoption as reported by athletic trainers (*n* = 529) across time points. Values presented as n(%) for those who responded they are adopting a policy.

Components of Best Practice Standards Q: My School’s Policies and Procedures	Aggregate (Both Fall + Spring)	Fall	Spring
**Include exertional heat illness (prevention and treatment) (total sample sizes for aggregate = 529, fall = 311, spring = 218)**	368 (69.6)	217 (69.8)	151 (69.3)
**Are based on environmental conditions measured by an on-site wet-bulb globe thermometer (total sample sizes for aggregate = 364, fall = 214, spring = 150)**	205 (56.31)	115 (53.7)	90 (60.0)
**Are based on environmental conditions that are specific to my region of the country (regionally specific) (total sample sizes for aggregate = 362, fall = 212, spring = 150)**	300 (82.9)	171 (80.7)	129 (86.0)
**Include a minimum of 4 levels of modification, including the modification of practice time based on environmental conditions (total sample sizes for aggregate = 364, fall = 215, spring = 149)**	284 (78.0)	171 (79.5)	113 (75.8)
**Include modification of work: rest ratios based on environmental conditions (total sample sizes for aggregate = 365, fall = 215, spring = 150)**	283 (77.5)	168 (78.1)	115 (76.7)
**Include modification of protective equipment (if applicable to sport) (total sample sizes for aggregate = 364, fall = 214, spring = 150)**	312 (85.7)	181 (84.6)	131 (87.3)
**Mention the use of shaded areas for rest breaks (total sample sizes for aggregate = 363, fall = 213, spring = 150)**	280 (77.1)	155 (72.8)	125 (83.3)

**Table 3:** PAPM stage from both fall and spring athletic trainer responses (aggregate). Values presented as n(%) for responses reported in each PAPM stage.

Q: My School’s Policies and Procedures…	*Unaware Needed*	*Unaware If Have*	*Decided Not to Act*	*Unengaged*	*Undecided*	*Decided to Act*	*Acting*	*Maintaining*
**Include exertional heat illness (prevention and treatment) (*n* = 515)**	14 (2.6)	26 (4.9)	8 (1.5)	34 (6.4)	42 (7.9)	37 (7)	39 (7.4)	329 (62.2)
**Are based on environmental conditions measured by an on-site wet-bulb globe thermometer (*n* = 351)**	13 (3.6)	25 (6.9)	36 (9.9)	30 (8.2)	36 (9.9)	16 (4.4)	32 (8.8)	176 (48.4)
**Are based on environmental conditions that are specific to my region of the country (regionally specific) (*n* = 351)**	11 (3)	13 (3.6)	7 (1.9)	7 (1.9)	14 (3.9)	10 (2.8)	34 (9.4)	266 (73.5)
**Include a minimum of four levels of modification, including the modification of practice time based on environmental conditions (*n* = 352)**	12 (3.3)	19 (5.2)	9 (2.5)	9 (2.5)	9 (2.5)	22 (6)	30 (8.2)	254 (69.8)
**Include modification of work: rest ratios based on environmental conditions (*n* = 347)**	18 (4.9)	20 (5.5)	9 (2.5)	9 (2.5)	12 (3.3)	14 (3.8)	33 (9)	250 (68.5)
**Include modification of protective equipment (if applicable to sport) (*n* = 354)**	10 (2.7)	8 (2.2)	8 (2.2)	6 (1.6)	8 (2.2)	12 (3.3)	30 (8.2)	282 (77.5)
**Mention the use of shaded areas for rest breaks (*n* = 350)**	13 (3.6)	15 (4.1)	12 (3.3)	20 (5.5)	11 (3)	12 (3.3)	26 (7.2)	254 (70)

**Table 4:** Facilitators and barriers to adoption of a comprehensive heat modification policy. Presented as n(%).

**Facilitators**	
Support from someone in an authoritative position (e.g., school leader, coach, nurse, etc.)	298 (56)
Having medical professional(s) (e.g., athletic trainer) at the school	260 (48.9)
State mandate from the high school athletics association	261 (49.1)
State legislation to mandate this policy	261 (49.1)
Seeing how other schools/programs implement this policy	200 (37.6)
Model policy that can be adopted	190 (35.7)
School stakeholders believing sport safety is important and buying into these policies	185 (34.8)
Nothing would make it easier	31 (5.8)
Training	17 (3.2)
**Barriers**	
No barriers encountered	232 (43.6)
Resistance or apprehension from head coaches to modify practices	179 (33.6)
My school’s AT is not full-time	60 (11.3)
My school would need more information, assistance, etc. in order to implement all of the heat modification guidelines	57 (10.7)
Resistance or apprehension from parents or legal guardians to modify practices	37 (7)
It’s not hot enough where I live; we have difficulty seeing the need for this	31 (5.8)
Liability	29 (5.5)
My school does not have the time to educate the parents or legal guardians on the importance of this policy	21 (3.9)
My school does not have the time to train the coaches and school personnel on how to implement this policy	20 (3.8)
We don’t think this policy is as important as other topics	14 (2.6)
We are located in a location that makes it difficult for EMS to get to us	5 (0.9)

## Supplementary Materials

The following are available online at https://www.mdpi.com/1010-660X/56/10/486/s1, supplementary file: Questionnaire.

## References

[1] Meehl G.A. (2004). More Intense, More Frequent, and Longer Lasting Heat Waves in the 21st Century. Science.

[2] Yeargin S.W., Dompier T.P., Casa D.J., Hirschhorn R.M., Kerr Z.Y. (2019). Epidemiology of Exertional Heat Illnesses in National Collegiate Athletic Association Athletes During the 2009–2010 Through 2014–2015 Academic Years. J. Athl. Train..

[3] Kerr Z.Y., Register-Mihalik J.K., Pryor R.R., Pierpoint L.A., Scarneo S.E., Adams W.M., Kucera K.L., Casa D.J., Marshall S.W. (2019). The Association between Mandated Preseason Heat Acclimatization Guidelines and Exertional Heat Illness during Preseason High School American Football Practices. Environ. Health Perspect..

[4] Kerr Z.Y., Casa D.J., Marshall S.W., Comstock R.D. (2013). Epidemiology of Exertional Heat Illness Among U.S. High School Athletes. Am. J. Prev. Med..

[5] Hosokawa Y., Casa D.J., Trtanj J.M., Belval L.N., Deuster P.A., Giltz S.M., Grundstein A.J., Hawkins M.D., Huggins R.A., Jacklitsch B. (2019). Activity modification in heat: Critical assessment of guidelines across athletic, occupational, and military settings in the USA. Int. J. Biometeorol..

[6] Cooper E.R., Grundstein A.J., Miles J.D., Ferrara M.S., Curry P., Casa D.J., Hosokawa Y. (2020). Heat Policy Revision for Georgia High School Football Practices Based on Data-Driven Research. J. Athl. Train..

[7] Budd G.M. (2008). Wet-bulb globe temperature (WBGT)—Its history and its limitations. J. Sci. Med. Sport.

[8] Minard D. (1961). Prevention of heat casualties in Marine Corps recruits. Period of 1955–60, with comparative incidence rates and climatic heat stresses in other training categories. Mil. Med..

[9] Armstrong L.E., Johnson E.C., Casa D.J., Ganio M.S., McDermott B.P., Yamamoto L.M., Lopez R.M., Emmanuel H. (2010). The American football uniform: Uncompensable heat stress and hyperthermic exhaustion. J. Athl. Train..

[10] Casa D., DeMartini J.K., Bergeron M.F., Csillan D., Eichner E.R., Lopez R.M., Ferrara M.S., Miller K.C., O’Connor F., Sawka M.N. (2015). National Athletic Trainers’ Association Position Statement: Exertional Heat Illnesses. J. Athl. Train..

[11] Tripp B., Vincent H.K., Bruner M., Smith M.S. (2020). Comparison of wet bulb globe temperature measured on-site vs estimated and the impact on activity modification in high school football. Int. J. Biometeorol..

[12] Pryor J.L., Pryor R.R., Grundstein A., Casa D.J. (2017). The Heat Strain of Various Athletic Surfaces: A Comparison Between Observed and Modeled Wet-Bulb Globe Temperatures. J. Athl. Train..

[13] Grundstein A., Williams C., Phan M., Cooper E. (2015). Regional heat safety thresholds for athletics in the contiguous United States. Appl. Geogr..

[14] Armstrong L.E., Casa D.J., Millard-Stafford M., Moran D.S., Pyne S.W., Roberts W.O., American College of Sports Medicine (2007). American College of Sports Medicine position stand. Exertional heat illness during training and competition. Med. Sci. Sport Exerc..

[15] Scarneo S.E., Distefano L.J., Stearns R.L., Register-Mihalik J.K., Denegar C.R., Casa D.J. (2019). Emergency Action Planning in Secondary-School Athletics: A Comprehensive Evaluation of Current Adoption of Best Practice Standards. J. Athl. Train..

[16] Scarneo-Miller S.E., DiStefano L.J., Register-Mihalik J.K., Stearns R.L., Denegar C.R., Casa D.J. (2019). Athletic Administrators Report of Emergency Action Plan Adoption in Secondary School Athletics. Appl. J. Sport Manag..

[17] Kerr Z.Y., Register-Mihalik J.K., Pryor R.R., Hosokawa Y., Scarneo-Miller S.E., Casa D.J. (2019). Compliance with the National Athletic Trainers’ Association Inter-Association Task Force Preseason Heat-Acclimatization Guidelines in High School Football. J. Athl. Train..

[18] Murata Y., Scarneo-Miller S.E., McMahon L., Casa D. (2020). Adoption of Emergency Action Plans in Secondary Schools: A Study of School Nurses’ Knowledge and Behavior. J. School Health.

[19] Scarneo-Miller S.E., DiStefano L.J., Singe S.M., Register-Mihalik J.K., Stearns R.L., Casa D.J. (2020). Emergency Action Plans in Secondary Schools: Barriers, Facilitators, and Social Determinants Affecting Implementation. J. Athl. Train..

[20] Adams W.M., Scarneo S.E., Casa D.J. (2017). State-Level Implementation of Health and Safety Policies to Prevent Sudden Death and Catastrophic Injuries Within Secondary School Athletics. Orthop. J. Sports Med..

[21] Athletic Training Locations and Services (ATLAS Project). https://ksi.uconn.edu/nata-atlas/.

[22] Crossway A., Rogers S.M., Nye E.A., Games K.E., Eberman L.E. (2019). Lesbian, Gay, Bisexual, Transgender, and Queer Athletic Trainers: Collegiate Student-Athletes’ Perceptions. J. Athl. Train..

[23] Casa D.J., Almquist J., Anderson S.A., Baker L., Bergeron M.F., Biagioli B., Boden B., Brenner J.S., Carroll M., Colgate B. (2013). The inter-association task force for preventing sudden death in secondary school athletics programs: Best-practices recommendations. J. Athl. Train..

[24] Casa D.J., Guskiewicz K.M., Anderson S.A., Courson R.W., Heck J.F., Jimenez C.C., McDermott B.P., Miller M.G., Stearns R.L., Swartz E.E. (2012). National athletic trainers’ association position statement: Preventing sudden death in sports. J. Athl. Train..

[25] Weinstein N.D., Sandman P.M., Blalock S.J. (2008). The Precaution Adoption Process Model. Health Behavior and Health Education.

[26] Binkley H.M., Beckett J., Casa D.J., Kleiner D.M., Plummer P.E. (2002). National Athletic Trainers’ Association Position Statement: Exertional Heat Illnesses. J. Athl. Train..

[27] Grundstein A., Cooper E. (2018). Assessment of the Australian Bureau of Meteorology wet bulb globe temperature model using weather station data. Int. J. Biometeorol..

[28] Cooper E.R., Ferrara M.S., Casa D.J., Powell J.W., Broglio S.P., Resch J.E., Courson R.W. (2016). Exertional Heat Illness in American Football Players: When Is the Risk Greatest?. J. Athl. Train..

[29] Grundstein A.J., Hosokawa Y., Casa D.J. (2018). Fatal Exertional Heat Stroke and American Football Players: The Need for Regional Heat-Safety Guidelines. J. Athl. Train..

