# Supplementary material for: Exertional Heat Illness Preparedness Strategies: Environmental Monitoring Policies in United States High Schools
Source: Medicina (Kaunas). 2020 Sep 23;56(10):486. doi: 10.3390/medicina56100486 (PMC7597963; doi:10.3390/medicina56100486)
Supplement: Supplementary file 1 [file medicina-56-00486-s001.pdf]

Supplement – Questionnaire

Do you currently work in a secondary school?

- o Yes
- o No

What is your current role or position at your high school?

- o Principal/Headmaster
- o Athletic Director
- o Head Coach
- o Assistant Coach
- o Nurse
- o Athletic Trainer
- o Parent of a Student- Athlete
- o Student-Athlete

Age: ____

How many students are enrolled at your high school?

____

How many years have you served in your role at your school?

- o Less than 1 year
- o 1-5 years
- o 6-10 years
- o 11-15 years
- o 15 or more years

How many years have you worked in your profession?

- o Less than 1 year
- o 1-5 years
- o 6-10 years
- o 11-15 years
- o 15 or more years

For each component, please select the category that best describes your high school's current written policies and procedures.

My school has policies and procedures on...

	My high school was **not aware** we needed to have this written policy	**I do not know** if my high school has this written policy	My high school is aware of this but has **not considered** creating this written policy	My high school is aware of this and is **considering** creating this written policy	My high school is aware of this policy, but has **decided not to** have this written policy	My high school is aware of this and **planning to create** this written policy within the next 6 months	My high school created this written policy **in the past 6 months**	My high school has had this written policy **for longer than 6 months**
Exertional Heat Illness (prevention and treatment)								
Is based on environmental conditions measured by an **on-site Wet-Bulb Globe Thermometer**								
Is based on environmental conditions that are **specific to my region of the country** (regionally specific)								
Includes a minimum of **4 levels of modification**, including the modification of practice time based on environmental conditions								

Includes modification of **work:rest ratios** based on environmental conditions	○	○	○	○	○	○	○	○
Includes modification of **protective equipment** (if applicable to sport)	○	○	○	○	○	○	○	○
Mentions the use of **shaded areas** for rest breaks	○	○	○	○	○	○	○	○

If your school has heat modification policies, who is the primary person who oversees the process (checks environmental conditions each day, informs coaches, etc.) to ensure that the progression is followed? (Select all that apply if this is a collaboration of efforts)

- o Principal/Head Master
- o Vice-Principal/Assistant Headmaster
- o Athletic Director
- o School Nurse
- o Athletic Trainer
- o Strength and Conditioning Coach
- o Head Coach Assistant Coach
- o My school has a heat modification policy, but I do not know who oversees the process
- o My school does not have a heat modification policy in place

Our school modifies activities in the heat based on:

- o On-site device that measures Wet Bulb Globe Temperature
- o On-site device that measures Heat Index
- o Estimation of WBGT based on on-site device that measures Heat Index
- o Website/phone application for Wet Bulb Globe Temperature
- o Website/phone application for Heat Index (or "Feels Like"/"Real Feel")
- o Estimation of WBGT based on website/phone application heat index
- o Temperature
- o My school does not have a heat modification policy
- o Other (please describe) ____

Which, if any, of the following do you foresee OR which, if any of the following did you encounter as <u>barriers</u> to your school's ability to implement a comprehensive <u>heat modification</u> <u>policy</u>? <u>Please check all that apply.</u>

- o Resistance or apprehension from head coaches to modify practices
- o Resistance or apprehension from parents or legal guardians to modify practices
- o Financial limitations
- o My school does not have the time to train the coaches and school personnel on how to implement this policy
- o My school does not have the time to educate the parents or legal guardians on the importance of this policy
- o My school would need more information, assistance, etc. in order to implement all of the heat modification guidelines
- o My school does not have an AT My school's AT is not full-time
- o It's not hot enough where I live, we have difficulty seeing the need for this
- o We are located in a location that makes it difficult for EMS to get to us
- o Liability
- o We don't think this policy is as important as other topics
- o No barriers encountered
- o Other: ____

Which, if any, of the following do you foresee OR which, if any, of the following did you Select all of the following that you feel would make it <u>easier</u> OR did make it easier to adopt a <u>heat modification policy</u>.

- ○ Having medical professional(s) (i.e. athletic trainer) at the school
- ○ Support from someone in an authoritative position (i.e.school leader, coach, nurse, etc.)
- ○ Seeing how other schools/programs implement this policy
- ○ State mandate from the high school athletics association
- ○ State legislation to mandate this policy
- ○ School stakeholders believing sport safety is important and buying into these policies
- ○ Model policy that can be adopted
- ○ Training
- ○ Nothing would make it easier
- ○ Other: ____
